# Methyl 2-(3-chloro-2-methyl­anilino)pyridine-3-carboxyl­ate

**DOI:** 10.1107/S2414314621005393

**Published:** 2021-05-25

**Authors:** Xing Yang, Sihui Long

**Affiliations:** a School of Chemical Engineering and Pharmacy, Wuhan Institute of Technology, Wuhan, Hubei 430205, People’s Republic of China; University of Aberdeen, Scotland

**Keywords:** crystal structure, intra­molecular hydrogen bonding, π–π stacking, alcoholysis

## Abstract

The title compound, C_14_H_13_ClN_2_O_2_, was obtained during an attempt to grow single crystals of 4-acetyl­phenyl 2-[(3-chloro-2-methyl­phen­yl)amino]­nicotinate in methanol, and was probably generated by alcoholysis. Two intra­molecular hydrogen bonds are formed, one between the N—H group and the carbonyl O atom of the ester and the other between the *ortho sp* ^2^CH group of the benzene ring and the pyridine N atom. Aromatic π–π stacking [shortest centroid–centroid separation = 3.598 (2) Å] is observed in the extended structure.

## Abstract 2

The title compound arose from an unexpected alcoholysis of a prodrug by the methanol solvent.

## Graphical abstract

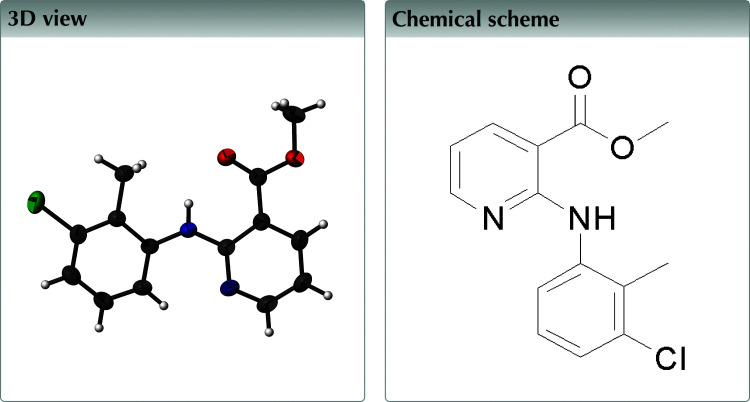

## Structure description

**Figure 1:**
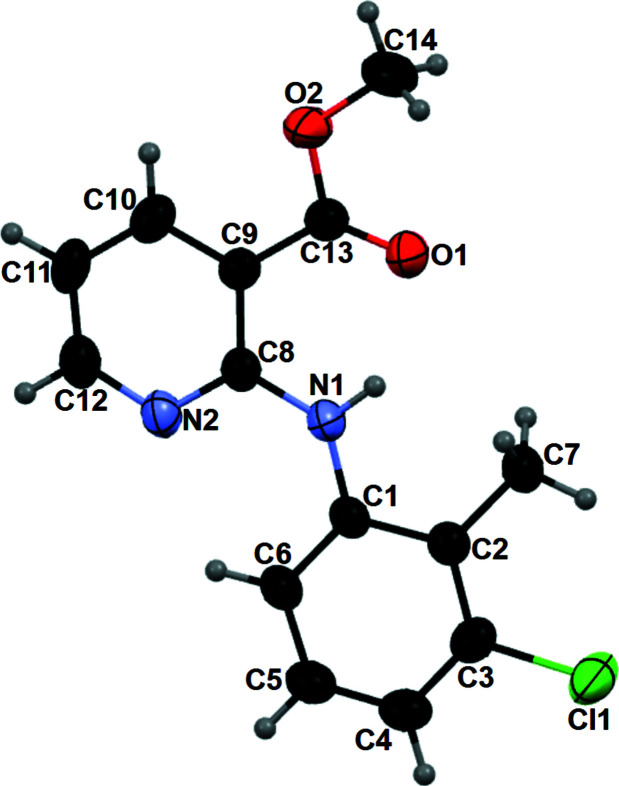
The mol­ecular structure of the title compound with displacement ellipsoids drawn at the 50% probability level.

**Figure 2:**
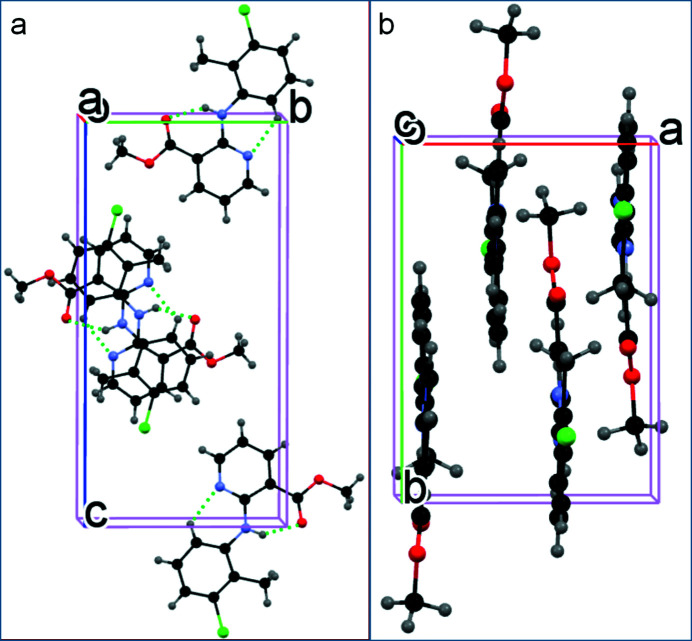
(*a*) Packing of the mol­ecules in the title compound viewed along [100] with the intra­molecular hydrogen bonds indicated by green dashed lines; (*b*) packing of the mol­ecules in the title compound viewed along [001].

The title compound (**I**) was first synthesized when preparing esters of anthranilic acid as possible analgesic and anti-inflammatory agents (Velingkar *et al.*, 2011 ▸). In our study, it was obtained during an effort to obtain single crystals of a codrug, 4-acetyl­phenyl 2-[(3-chloro-2-methyl­phen­yl)amino]­nicotinate, by slow evaporation in methanol. Colorless needles were harvested and structure determination by single-crystal X-ray diffraction revealed it to be the title compound: alcoholysis by methanol obviously led to the generation of **I**. The asymmetric unit of **I** consists of one mol­ecule with a near planar conformation as evidenced by the dihedral angle of 5.31 (1)° between the C1–C6 benzene and N2/C8–C12 pyridine rings (Fig. 1 ▸). Two intra­molecular hydrogen bonds are observed (Table 1 ▸), one between the N—H group and the carbonyl oxygen atom of the ester group with a donor–acceptor distance of 2.687 (3) Å, and the other between the *ortho sp* ^2^C—H grouping of the aniline ring and the pyridine N atom [2.895 (4) Å]: both of these close *S*(6) rings. The cohesion of the crystal structure is ensured by aromatic π–π stacking between the benzene and pyridine rings [shortest centroid–centroid separation = 3.598 (2) Å] and hydro­phobic inter­actions (Fig. 2 ▸).

## Synthesis and crystallization

**Figure 3:**
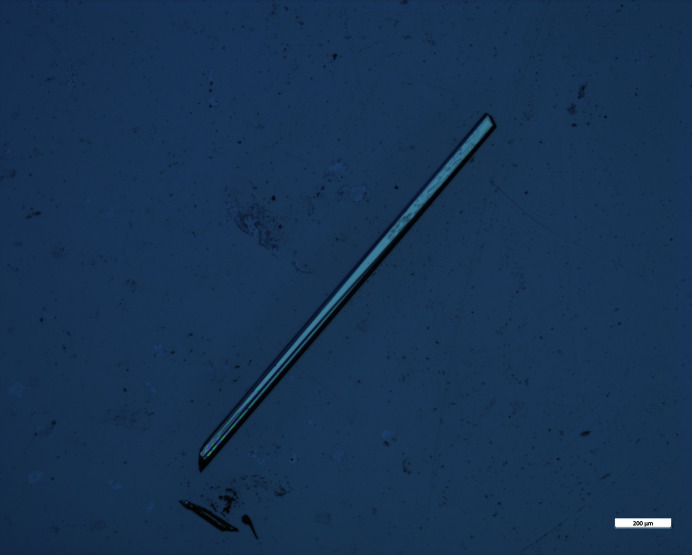
A representative crystal of I.

**Figure 4:**
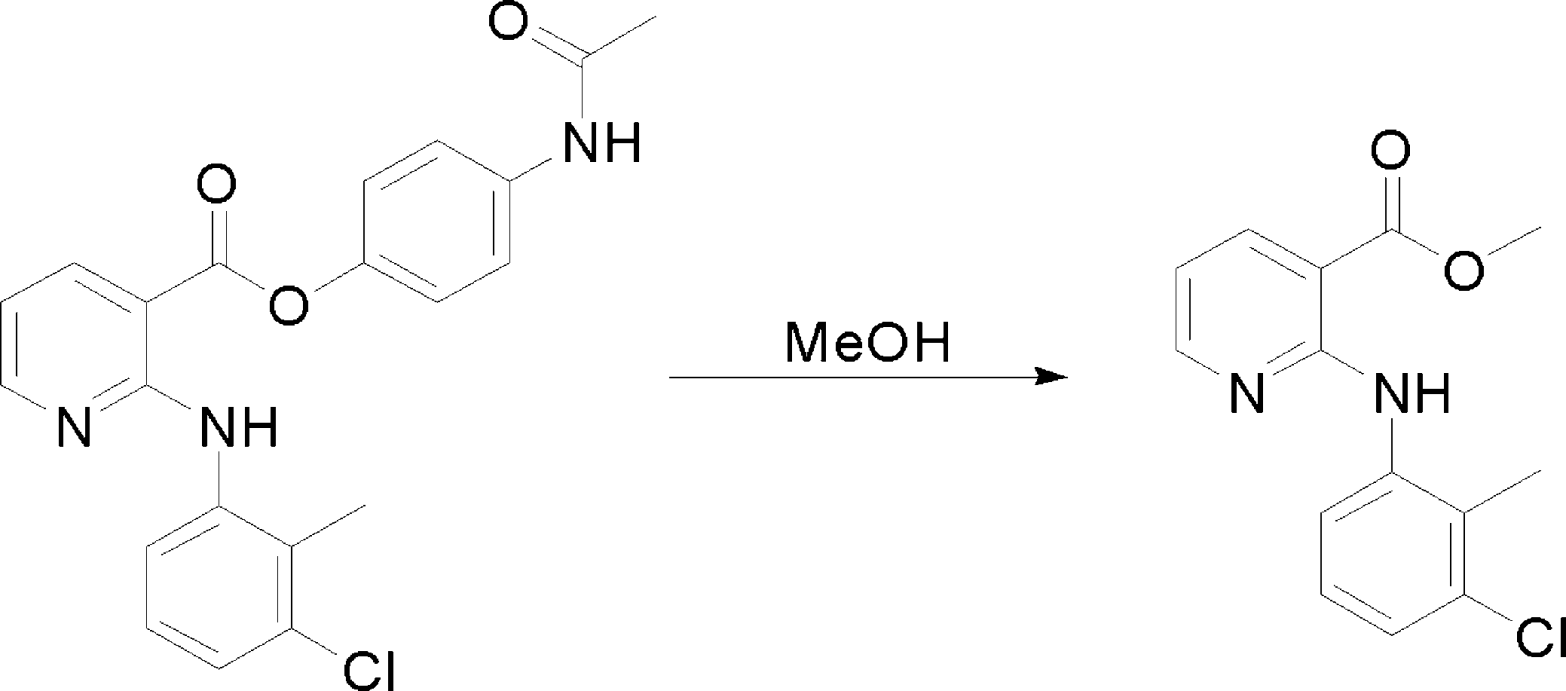
Reaction scheme.

4-Acetyl­phenyl 2-[(3-chloro-2-methyl­phen­yl)amino]­nicotin­ate, synthesized by a condensation reaction between clonixin and paracetamol (Gupta & Moorthy, 2007 ▸), was dissolved in HPLC grade methanol to make a saturated solution. The solution underwent slow evaporation at room temperatures and colorless needle-shaped crystals of the title compound (Fig. 3 ▸) were harvested after about a week. Alcoholysis by methanol likely resulted in the formation of the title compound (Fig. 4 ▸).

## Refinement

Crystal data, data collection and structure refinement details are summarized in Table 2 ▸.

## Supplementary Material

Crystal structure: contains datablock(s) global, I. DOI: 10.1107/S2414314621005393/hb4383sup1.cif

Structure factors: contains datablock(s) I. DOI: 10.1107/S2414314621005393/hb4383Isup2.hkl

Supporting information file. DOI: 10.1107/S2414314621005393/hb4383Isup3.cml

CCDC reference: 2085247

Additional supporting information:  crystallographic information; 3D view; checkCIF report

## Figures and Tables

**Table 1:** Hydrogen-bond geometry (Å, °)

*D*—H⋯*A*	*D*—H	H⋯*A*	*D*⋯*A*	*D*—H⋯*A*
N1—H1⋯O1	0.86	1.96	2.687 (3)	142
C6—H6⋯N2	0.93	2.28	2.895 (4)	123

**Table 2:** Experimental details

Crystal data	
Chemical formula	C_14_H_13_ClN_2_O_2_
*M*_r_	276.71
Crystal system, space group	Orthorhombic, *P*2_1_2_1_2_1_
Temperature (K)	296
*a*, *b*, *c* (Å)	6.919 (2), 9.653 (3), 19.319 (6)
*V* (Å^3^)	1290.4 (6)
*Z*	4
Radiation type	Mo *K*α
μ (mm^−1^)	0.30
Crystal size (mm)	0.2 × 0.2 × 0.1

Data collection	
Diffractometer	Bruker APEXII CCD
Absorption correction	Multi-scan (*SADABS*; Bruker, 2013 ▸)
*T*_min_, *T*_max_	0.544, 0.746
No. of measured, independent and observed [*I* > 2σ(*I*)] reflections	7991, 4200, 2872
*R*_int_	0.031
(sin θ/λ)_max_ (Å^−1^)	0.746

Refinement	
*R*[*F* ^2^ > 2σ(*F* ^2^)], *wR*(*F* ^2^), *S*	0.048, 0.144, 1.01
No. of reflections	4200
No. of parameters	174
H-atom treatment	H-atom parameters constrained
Δρ_max_, Δρ_min_ (e Å^−3^)	0.23, −0.39
Absolute structure	Flack *x* determined using 963 quotients [(*I* ^+^)−(*I* ^−^)]/[(*I* ^+^)+(*I* ^−^)] (Parsons *et al.*, 2013 ▸)
Absolute structure parameter	−0.02 (4)

Computer programs: *APEX2* and *SAINT* (Bruker, 2013 ▸), *SHELXS* (Sheldrick, 2015*b* ▸), *SHELXL* (Sheldrick, 2015*a* ▸) and *OLEX2* (Dolomanov *et al.*, 2009 ▸).

## full crystallographic data

### Crystal data

**Table:**

C_14_H_13_ClN_2_O_2_	*D*_x_ = 1.424 Mg m^−^^3^
*M**_r_* = 276.71	Mo *K*α radiation, λ = 0.71073 Å
Orthorhombic, *P*2_1_2_1_2_1_	Cell parameters from 2539 reflections
*a* = 6.919 (2) Å	θ = 2.4–31.0°
*b* = 9.653 (3) Å	µ = 0.30 mm^−^^1^
*c* = 19.319 (6) Å	*T* = 296 K
*V* = 1290.4 (6) Å^3^	Block, yellow
*Z* = 4	0.2 × 0.2 × 0.1 mm
*F*(000) = 576	

### Data collection

**Table:**

Bruker APEXII CCD diffractometer	2872 reflections with *I* > 2σ(*I*)
φ and ω scans	*R*_int_ = 0.031
Absorption correction: multi-scan (SADABS; Bruker, 2013)	θ_max_ = 32.0°, θ_min_ = 2.1°
*T*_min_ = 0.544, *T*_max_ = 0.746	*h* = −9→10
7991 measured reflections	*k* = −11→13
4200 independent reflections	*l* = −14→28

### Refinement

**Table:**

Refinement on *F*^2^	Hydrogen site location: inferred from neighbouring sites
Least-squares matrix: full	H-atom parameters constrained
*R*[*F*^2^ > 2σ(*F*^2^)] = 0.048	*w* = 1/[σ^2^(*F*_o_^2^) + (0.0816*P*)^2^] where *P* = (*F*_o_^2^ + 2*F*_c_^2^)/3
*wR*(*F*^2^) = 0.144	(Δ/σ)_max_ < 0.001
*S* = 1.01	Δρ_max_ = 0.23 e Å^−^^3^
4200 reflections	Δρ_min_ = −0.38 e Å^−^^3^
174 parameters	Absolute structure: Flack *x* determined using 963 quotients [(*I*^+^)-(*I*^-^)]/[(*I*^+^)+(*I*^-^)] (Parsons *et al.*, 2013)
0 restraints	Absolute structure parameter: −0.02 (4)
Primary atom site location: structure-invariant direct methods	

### Special details

**Table:**

Geometry. All esds (except the esd in the dihedral angle between two l.s. planes) are estimated using the full covariance matrix. The cell esds are taken into account individually in the estimation of esds in distances, angles and torsion angles; correlations between esds in cell parameters are only used when they are defined by crystal symmetry. An approximate (isotropic) treatment of cell esds is used for estimating esds involving l.s. planes.
Refinement. H atoms were located in difference Fourier maps and subsequently placed in idealized positions with C—H = 0.95–0.96 and N—H = 0.86 Å: *U*_iso_(H) values were constrained to 1.2*U*_eq_(C,*N*) or 1.5*U*_eq_(methyl C).

### Fractional atomic coordinates and isotropic or equivalent isotropic displacement parameters (Å^2^)

**Table:**

	*x*	*y*	*z*	*U*_iso_*/*U*_eq_	
Cl1	0.36941 (14)	0.30941 (9)	0.76342 (4)	0.0564 (2)	
O1	0.3954 (4)	−0.07829 (19)	0.49264 (10)	0.0550 (6)	
O2	0.4138 (4)	−0.16139 (19)	0.38542 (11)	0.0588 (7)	
N1	0.3847 (4)	0.19868 (19)	0.50569 (10)	0.0375 (4)	
H1	0.3865	0.1159	0.5221	0.045*	
N2	0.3731 (4)	0.3270 (2)	0.40324 (11)	0.0396 (5)	
C1	0.3845 (4)	0.3013 (2)	0.55605 (12)	0.0325 (4)	
C2	0.3765 (4)	0.2557 (2)	0.62562 (12)	0.0330 (5)	
C3	0.3778 (4)	0.3568 (3)	0.67663 (13)	0.0377 (5)	
C4	0.3860 (5)	0.4979 (3)	0.66203 (15)	0.0442 (6)	
H4	0.3869	0.5631	0.6975	0.053*	
C5	0.3927 (5)	0.5379 (3)	0.59459 (16)	0.0468 (7)	
H5	0.3978	0.6319	0.5842	0.056*	
C6	0.3921 (5)	0.4434 (2)	0.54119 (14)	0.0416 (6)	
H6	0.3968	0.4737	0.4955	0.050*	
C7	0.3687 (6)	0.1035 (3)	0.64212 (15)	0.0439 (6)	
H7A	0.2541	0.0639	0.6222	0.066*	
H7B	0.3664	0.0910	0.6914	0.066*	
H7C	0.4806	0.0584	0.6232	0.066*	
C8	0.3826 (4)	0.2042 (2)	0.43491 (12)	0.0321 (4)	
C9	0.3880 (4)	0.0779 (2)	0.39658 (12)	0.0338 (5)	
C10	0.3839 (5)	0.0864 (3)	0.32512 (13)	0.0416 (6)	
H10	0.3879	0.0061	0.2986	0.050*	
C11	0.3739 (5)	0.2146 (3)	0.29305 (13)	0.0445 (6)	
H11	0.3708	0.2224	0.2451	0.053*	
C12	0.3689 (5)	0.3283 (3)	0.33432 (14)	0.0439 (6)	
H12	0.3619	0.4142	0.3127	0.053*	
C13	0.3982 (5)	−0.0571 (3)	0.43094 (14)	0.0403 (6)	
C14	0.4245 (8)	−0.2972 (3)	0.4152 (2)	0.0734 (13)	
H14A	0.4497	−0.3638	0.3794	0.110*	
H14B	0.3041	−0.3189	0.4374	0.110*	
H14C	0.5269	−0.2999	0.4487	0.110*	

### Atomic displacement parameters (Å^2^)

**Table:**

	*U*^11^	*U*^22^	*U*^33^	*U*^12^	*U*^13^	*U*^23^
Cl1	0.0711 (5)	0.0639 (5)	0.0342 (3)	0.0059 (5)	0.0011 (3)	−0.0033 (3)
O1	0.0918 (18)	0.0370 (9)	0.0362 (10)	−0.0004 (12)	−0.0012 (12)	0.0045 (7)
O2	0.103 (2)	0.0316 (9)	0.0414 (11)	0.0015 (11)	−0.0110 (12)	−0.0021 (8)
N1	0.0511 (12)	0.0297 (8)	0.0318 (9)	−0.0002 (12)	−0.0003 (10)	0.0035 (8)
N2	0.0447 (11)	0.0365 (10)	0.0377 (11)	0.0001 (11)	−0.0010 (11)	0.0080 (8)
C1	0.0316 (10)	0.0301 (10)	0.0358 (11)	−0.0001 (11)	−0.0004 (11)	0.0036 (9)
C2	0.0297 (11)	0.0329 (10)	0.0362 (12)	0.0010 (10)	−0.0007 (11)	0.0018 (8)
C3	0.0326 (12)	0.0451 (13)	0.0353 (12)	0.0040 (12)	0.0002 (12)	−0.0033 (10)
C4	0.0447 (15)	0.0384 (12)	0.0494 (15)	0.0013 (13)	−0.0004 (14)	−0.0085 (10)
C5	0.0550 (17)	0.0327 (11)	0.0527 (17)	−0.0012 (13)	0.0016 (16)	−0.0038 (11)
C6	0.0523 (15)	0.0307 (10)	0.0417 (13)	0.0001 (12)	−0.0005 (14)	0.0052 (9)
C7	0.0562 (16)	0.0372 (11)	0.0383 (13)	0.0008 (14)	−0.0001 (15)	0.0068 (10)
C8	0.0280 (10)	0.0353 (10)	0.0329 (10)	0.0003 (12)	0.0011 (10)	0.0026 (9)
C9	0.0319 (11)	0.0370 (10)	0.0326 (11)	−0.0015 (11)	−0.0012 (11)	0.0009 (9)
C10	0.0428 (14)	0.0505 (14)	0.0314 (12)	0.0037 (14)	−0.0007 (12)	−0.0014 (11)
C11	0.0481 (14)	0.0561 (15)	0.0292 (12)	0.0037 (16)	−0.0002 (12)	0.0091 (11)
C12	0.0454 (14)	0.0450 (14)	0.0415 (14)	0.0030 (15)	−0.0010 (13)	0.0122 (10)
C13	0.0482 (16)	0.0359 (11)	0.0368 (13)	−0.0026 (12)	−0.0025 (12)	−0.0012 (10)
C14	0.129 (4)	0.0296 (13)	0.061 (2)	0.0016 (18)	−0.019 (2)	0.0000 (14)

### Geometric parameters (Å, º)

**Table:**

Cl1—C3	1.739 (3)	C5—C6	1.377 (4)
O1—C13	1.210 (3)	C6—H6	0.9300
O2—C13	1.341 (3)	C7—H7A	0.9600
O2—C14	1.434 (3)	C7—H7B	0.9600
N1—H1	0.8600	C7—H7C	0.9600
N1—C1	1.388 (3)	C8—C9	1.428 (3)
N1—C8	1.369 (3)	C9—C10	1.383 (3)
N2—C8	1.335 (3)	C9—C13	1.464 (3)
N2—C12	1.332 (3)	C10—H10	0.9300
C1—C2	1.415 (3)	C10—C11	1.386 (4)
C1—C6	1.403 (3)	C11—H11	0.9300
C2—C3	1.387 (3)	C11—C12	1.357 (4)
C2—C7	1.504 (3)	C12—H12	0.9300
C3—C4	1.391 (4)	C14—H14A	0.9600
C4—H4	0.9300	C14—H14B	0.9600
C4—C5	1.360 (4)	C14—H14C	0.9600
C5—H5	0.9300		
			
C13—O2—C14	115.3 (2)	H7A—C7—H7C	109.5
C1—N1—H1	113.9	H7B—C7—H7C	109.5
C8—N1—H1	113.9	N1—C8—C9	119.0 (2)
C8—N1—C1	132.2 (2)	N2—C8—N1	119.5 (2)
C12—N2—C8	117.9 (2)	N2—C8—C9	121.5 (2)
N1—C1—C2	116.3 (2)	C8—C9—C13	121.8 (2)
N1—C1—C6	123.7 (2)	C10—C9—C8	117.8 (2)
C6—C1—C2	120.0 (2)	C10—C9—C13	120.4 (2)
C1—C2—C7	120.4 (2)	C9—C10—H10	120.0
C3—C2—C1	117.1 (2)	C9—C10—C11	120.0 (2)
C3—C2—C7	122.5 (2)	C11—C10—H10	120.0
C2—C3—Cl1	119.9 (2)	C10—C11—H11	121.3
C2—C3—C4	123.0 (2)	C12—C11—C10	117.4 (2)
C4—C3—Cl1	117.0 (2)	C12—C11—H11	121.3
C3—C4—H4	120.9	N2—C12—C11	125.4 (2)
C5—C4—C3	118.3 (2)	N2—C12—H12	117.3
C5—C4—H4	120.9	C11—C12—H12	117.3
C4—C5—H5	119.0	O1—C13—O2	121.4 (2)
C4—C5—C6	122.0 (2)	O1—C13—C9	126.6 (2)
C6—C5—H5	119.0	O2—C13—C9	112.0 (2)
C1—C6—H6	120.2	O2—C14—H14A	109.5
C5—C6—C1	119.6 (3)	O2—C14—H14B	109.5
C5—C6—H6	120.2	O2—C14—H14C	109.5
C2—C7—H7A	109.5	H14A—C14—H14B	109.5
C2—C7—H7B	109.5	H14A—C14—H14C	109.5
C2—C7—H7C	109.5	H14B—C14—H14C	109.5
H7A—C7—H7B	109.5		
			
Cl1—C3—C4—C5	180.0 (3)	C7—C2—C3—Cl1	−0.2 (4)
N1—C1—C2—C3	179.5 (3)	C7—C2—C3—C4	179.6 (3)
N1—C1—C2—C7	0.0 (4)	C8—N1—C1—C2	176.7 (3)
N1—C1—C6—C5	−179.5 (3)	C8—N1—C1—C6	−3.5 (5)
N1—C8—C9—C10	179.4 (3)	C8—N2—C12—C11	−0.2 (5)
N1—C8—C9—C13	−0.8 (4)	C8—C9—C10—C11	−0.3 (4)
N2—C8—C9—C10	0.3 (4)	C8—C9—C13—O1	3.2 (5)
N2—C8—C9—C13	−179.9 (3)	C8—C9—C13—O2	−176.2 (3)
C1—N1—C8—N2	−2.3 (5)	C9—C10—C11—C12	0.1 (5)
C1—N1—C8—C9	178.6 (3)	C10—C9—C13—O1	−177.0 (3)
C1—C2—C3—Cl1	−179.7 (2)	C10—C9—C13—O2	3.6 (4)
C1—C2—C3—C4	0.2 (4)	C10—C11—C12—N2	0.1 (5)
C2—C1—C6—C5	0.3 (4)	C12—N2—C8—N1	−179.2 (3)
C2—C3—C4—C5	0.1 (5)	C12—N2—C8—C9	0.0 (4)
C3—C4—C5—C6	−0.2 (5)	C13—C9—C10—C11	179.9 (3)
C4—C5—C6—C1	0.0 (5)	C14—O2—C13—O1	0.6 (5)
C6—C1—C2—C3	−0.3 (4)	C14—O2—C13—C9	−180.0 (3)
C6—C1—C2—C7	−179.8 (3)		

### Hydrogen-bond geometry (Å, º)

**Table:**

*D*—H···*A*	*D*—H	H···*A*	*D*···*A*	*D*—H···*A*
N1—H1···O1	0.86	1.96	2.687 (3)	142
C6—H6···N2	0.93	2.28	2.895 (4)	123
